# Reversible magnetic spiral domain

**DOI:** 10.1038/s41598-021-00016-z

**Published:** 2021-10-25

**Authors:** Kyoung-Woong Moon, Seungmo Yang, Chanyong Hwang

**Affiliations:** Quantum Spin Team, Korea Research Institute of Standards and Science, Daejeon, 34113 Republic of Korea

**Keywords:** Magnetic properties and materials, Spintronics

## Abstract

The various spiral structures that exist in nature inspire humanity because of their morphological beauty, and spiral structures are used in various fields, including architecture, engineering, and art. Spiral structures have their own winding directions, and in most spirals, it is difficult to reverse the predetermined winding direction. Here, we show that a rotating spiral exists in magnetic systems for which the winding direction can be easily reversed. A magnetization vector basically has a spiral motion combining a precessional and a damping motion. The application of these basic mechanics to a system composed of magnetic vectors that are affected by a radial current and the Dzyaloshinskii–Moriya interaction forms the rotating magnetic spiral. The winding direction of the magnetic spiral has its own stability, but the direction can be changed using an external magnetic field. This magnetic spiral has a finite size, and the magnetic domain is destroyed at the edge of the spiral, which can create magnetic skyrmions.

## Introduction

There are many spiral structures in nature that are formed by various causes^1^. One of the most familiar examples, which is also huge in extent, is a spiral galaxy^1–3^. Several theories have been raised about the creation of spiral arms, such as the density wave theory and tidal interaction^1–3^. A tropical cyclone (hurricane, typhoon, cyclone) is probably the largest spiral that directly affects humans^1,4,5^. Warm sea level temperatures and strong rising air currents are known to be important causes of tropical cyclones, and the direction of spiral rotation is determined by the Coriolis effect^1,4,5^. Apart from these giant spirals, spirals also exist on the atomic scale in crystalline growth^1,6,7^. In living things, gastropod shells have spiral shapes because it is the most convenient way to keep growing in size while maintaining the same shape^1^. In several works, it was found that the spiral direction of the shell is determined from the early stages of embryonic development^1,8,9^. In plants, the arrangement of sunflower seeds and patterns of pine cones display a spiral structure related to the Fibonacci numbers^1,10^. These spiral structures have inspired humanity due to their morphological beauty and stability, and have been used since ancient times in the fields of art, music, literature, design, engineering, and architecture^1,11,12^. These various spirals are formed with different origins, but all spiral structures have a common characteristic of winding directions (clockwise and counterclockwise). We can define the winding direction as the direction of rotation when moving from outside to centre along the spiral arm. Note that, once the direction of this winding is established, it is impossible to reverse this.

In this paper, we show that rotating spiral structures can also be formed in magnetic systems, which can be deduced and verified in the laboratory. Spirals that are present in magnetic systems have distinct characteristics from the various spirals described earlier. That is the winding direction of the magnetic spiral can be changed using an external magnetic field. It is notable that magnetization states of spiral vortices and solutions for stationary modes were discovered by Borisov et al.^13^. An important difference from this previous report is that our results account for the dynamic phenomena of rotating spiral arms. Numerical simulations and analytical calculations show in simple magnetic dynamics equations how the spiral structure occurs^14–21^. In addition to these basic characteristics, the possibility of forming a magnetic skyrmion^22–26^ was also confirmed using the magnetic spiral state. The magnetic skyrmion is considered an ideal information carrier for memory and logic devices due to its topological stability.

**Figure 1:**
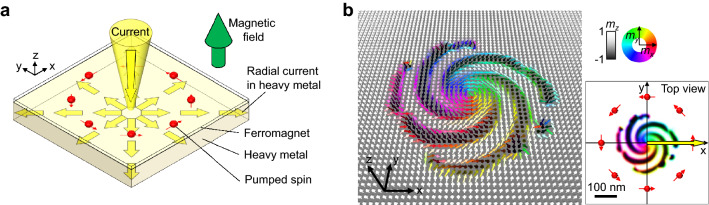
Magnetic spiral induced by radial current flow. (**a**), Vertically injected current into a local point of a ferromagnet/heavy metal structure and generation of a radial current (yellow arrows). The red arrows are the direction of the pumped spin. (**b**), The magnetization direction of the ferromagnetic material is indicated by coloured vectors. The rainbow colour represents the magnetization component on the *xy*-plane, and the grey scale represents the component in the *z*-direction. The yellow arrow indicates the area covered by the 1D model. Scale bar, 100 nm. The winding direction of this spiral is counterclockwise.

The situation covered in this paper is described schematically in Fig. 1. An electric current is injected vertically onto a local point of a conductor coated with a thin magnetic layer (1 nm thick) and forms a current that spreads radially. If the conductor material generates a sufficient spin Hall effect^27–30^ (such as a heavy metal), a spin is injected into the magnetic material in a specific direction. The direction of the pumped spin is determined by the cross product of the pumping direction ($+ \hat{z}$) and the current direction (radial) (red arrows in Fig. 1a). In this situation, if the magnetic material prefers $\pm \hat{z}$ and there is the Dzyaloshinskii–Moriya interaction, the current can generate a spiral-shaped magnetization state (Fig. 1b and see Supplementary Movie 1 for the spiral rotation). The reason for this state is explained below, starting from the magnetization vector.

### Stationary state of uniform magnetization

A magnetic system is composed of magnetization vectors. When a magnet is made of a single material or a miscible alloy, the magnetization vector has a constant size ($M_{{\text{S}}}$, saturation magnetization). By dividing the magnetization vector by $M_{{\text{S}}}$, the magnetization state can be described with unit vectors ${\mathbf{m}} = \left( {m_{x} ,{ }m_{y} ,m_{z} } \right)$, where $m_{i}$ is the component along the $i$ axis and $m_{x}^{2} + m_{y}^{2} + m_{z}^{2} = 1$. ${\mathbf{m}}$ can have different directions depending on location and time. Such magnetization dynamics are described by the Landau–Lifshitz–Gilbert (LLG) equation^14–21^. If we include the effect of spin–orbit torque (SOT) by the spin Hall effect, the basic form of the LLG equation becomes:

1 $${\dot{\mathbf{m}}} = - \gamma {\mathbf{m}} \times {\mathbf{H}}_{{{\text{eff}}}} + \alpha {\mathbf{m}} \times {\dot{\mathbf{m}}} - \gamma \tau_{{\text{d}}} {\mathbf{m}} \times \left( {{\mathbf{m}} \times {{\varvec{\upsigma}}}} \right).$$

$\gamma$ is the gyromagnetic ratio, ${\mathbf{H}}_{{{\text{eff}}}}$ is an effective field, $\alpha$ is a damping constant, $\tau_{{\text{d}}}$ is the magnitude of the damping-like SOT and ${{\varvec{\upsigma}}}$ is a unit vector of the pumped spin direction to ${\mathbf{m}}$. The right-hand terms of Eq. (1) describe, in order, the precessional motion by ${\mathbf{H}}_{{{\text{eff}}}}$, the phenomenological damping motion, and the damping-like motion by the pumped spin. A stationary state (${\dot{\mathbf{m}}} = 0$) can be achieved using,

2 $${\mathbf{m}} \times {\mathbf{H}}_{{{\text{eff}}}} = - \tau_{{\text{d}}} {\mathbf{m}} \times \left( {{\mathbf{m}} \times {{\varvec{\upsigma}}}} \right).$$

Let us consider the simplest situation of uniform magnetization (i.e. macrospin or single spin) and its stationary states^20,21,27^. This magnetization is held by a perpendicular magnetic anisotropy (PMA), which means ${\mathbf{H}}_{{{\text{eff}}}} = H_{k} m_{z} \hat{z}$ w﻿it﻿h an anisotropic field strength, $H_{k}$. For further simplification, only the case where $\tau_{{\text{d}}}$ is positive with ${{\varvec{\upsigma}}} = + \hat{y}$ is considered. We already know that when $\tau_{{\text{d}}} = 0$, ${\mathbf{m}}$ is aligned to $+ \hat{z}$ or $- \hat{z}$ due to the PMA^31,32^. These perpendicular magnetizations persist up to $\tau_{{\text{d}}} = H_{k} /2$ with a slightly rotated magnetization from $\pm \hat{z}$. This magnetization rotation follows $m_{x} m_{z} = - \tau_{{\text{d}}} /H_{k}$ and $m_{y} = 0$. When $\tau_{{\text{d}}} > H_{k} /2$, only ${\mathbf{m}} = + \hat{y}$ is possible (see Supplementary Note 1 for more detailed descriptions). From the explanation so far, it can be seen that a sudden jump in magnetization from ${\mathbf{m}} = \left( { \pm \frac{1}{\sqrt 2 },0, \mp \frac{1}{\sqrt 2 }} \right)$ to ${\mathbf{m}} = \left( {0,1,0} \right)$ occurs at |$\tau_{{\text{d}}} | = H_{k} /2$ with increasing $\tau_{{\text{d}}}$ or decreasing $H_{k}$.

### Formation of wave magnetization and translation of magnetization texture

**Figure 2:**
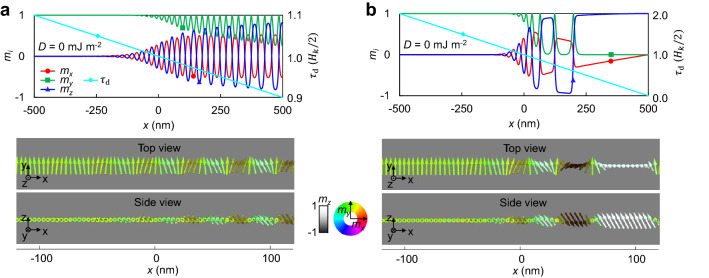
Formation of wave magnetization. (**a**), Stabilized magnetization state as a function of the position formed when $\tau_{{\text{d}}}$ changes slowly in position. (**b**), The state when $\tau_{{\text{d}}}$ changes rapidly.

We will now show that this magnetization jump creates a wave-shaped magnetization state. Considering the exchange interaction only in the *x*-direction^15,33,34^ (a unidimensional (1D) model) with the PMA, ${\mathbf{H}}_{{{\text{eff}}}} = A^{*} \partial^{2} {\mathbf{m}}/\partial x^{2} + H_{k} m_{z} \hat{z}$, where $A^{*}$ is $2A/\left( {\mu_{0} M_{{\text{S}}} } \right)$, $A$ is the exchange stiffness constant, and $\mu_{0}$ is the magnetic permeability^35–37^. $A > 0$ favours alignment of states between adjacent magnetic vectors. This 1D model means that only the yellow arrow area in Fig. 1b is considered. To show the effect of $A$, micromagnetic simulations using the MuMax3 program were performed^38^. It is assumed that $\tau_{{\text{d}}} = H_{k} /2$ is at the centre of the *x* position ($x = 0$ in Fig. 2a, b) and $\tau_{{\text{d}}}$ changes linearly along the *x*-axis. The characteristic wave-shaped magnetization state is stabilized by setting an initial random magnetization state and waiting for a stable state to be found according to the LLG equation (see Supplementary Note 2 for results starting from uniform magnetization and results with different cell sizes). The $+ \hat{y}$ state is stabilized in the position where $\tau_{{\text{d}}} > H_{k} /2$, (Fig. 2a, b), which is the same as the aforementioned uniform magnetization. However, small $m_{x}$ and $m_{z}$ components of the same amplitude occur in the vicinity of the $\tau_{{\text{d}}} = H_{k} /2$ area, and a complete perpendicular domain state is formed in the small $\tau_{{\text{d}}}$ area (see Supplementary Note 3 for more information about stable states). In addition, the wavelength near $\tau_{{\text{d}}} = H_{k} /2$ seems to be independent of the gradient of $\tau_{{\text{d}}}$, and the phases of the $m_{x}$ and $m_{z}$ oscillations are opposite to each other (Fig. 2a, b). This antiphase differs from normal spin waves with 90° phase difference between $m_{x}$ and $m_{z}$. Although the exact cause is currently unclear, we think this antiphase coupling could be described by the result of the previous section that the signs of $m_{x}$ and $m_{z}$ are interconnected due to $m_{x} m_{z} = - \tau_{{\text{d}}} /H_{k}$. From the simulation results, the wave magnetization near $\tau_{{\text{d}}} = H_{k} /2$ can be represented as ${\mathbf{m}} = \left( {X\sin kx,1, - X\sin kx} \right)$ with an amplitude $X$ (< < 1) and a wave number $k$. Using the stationary condition, the wave number is obtained as follows (see Supplementary Note 4 for more detailed procedures).

3 $$k^{2} = \frac{{H_{k} }}{{2A^{*} }} = \frac{{K_{z} }}{2A}.$$

Here, $K_{z}$ (= $\mu_{0} M_{{\text{S}}} H_{k} /2$) is the energy density of the PMA. Note that these waves do not move.

The Dzyaloshinskii–Moriya interaction^34,39–41^ (DMI) can move this wave. If the DMI is added to the 1D model, ${\mathbf{H}}_{{{\text{eff}}}} = A^{*} \partial^{2} {\mathbf{m}}/\partial x^{2} + D^{*} \hat{y} \times \left( {\partial {\mathbf{m}}/\partial x} \right) + H_{k} m_{z} \hat{z}$. Here, $D^{*}$ is $2D/\left( {\mu_{0} M_{{\text{S}}} } \right)$ and $D$ is the interfacial-type DMI energy density^28,36,38^. $D > 0$ prefers the (+ *z*)-(–*x*)-(–*z*)-(+ *x*) chiral states along the *x*-axis. Assuming that the moving magnetization state is ${\mathbf{m}} = \left( {X\sin \left( {kx - \omega t} \right),1, - X\sin \left( {kx - \omega t} \right)} \right)$ with an angular frequency $\omega$ and time $t$, the wave velocity ($V$) is obtained by performing the $\int_{0}^{2\pi /k} { \cdot \left( {\partial {\mathbf{m}}/\partial x} \right)dx}$ integration on both sides of Eq. (1). The result is (see Supplementary Note 5 for detailed procedures),

4 $$V = \frac{\omega }{k} = - \frac{{\gamma D^{*} }}{{\left( {1 + \alpha^{2} } \right)}} = - \frac{2\gamma D}{{\left( {1 + \alpha^{2} } \right)\mu_{0} M_{S} }}.{ }$$

**Figure 3:**
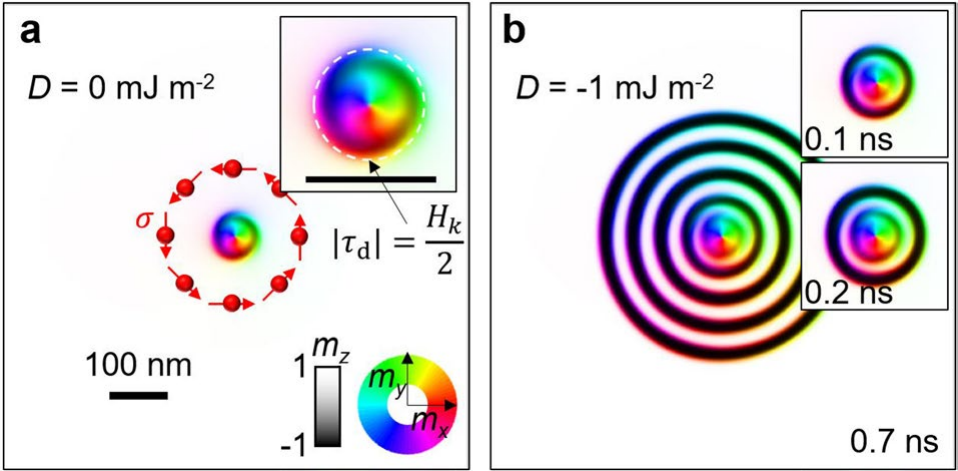
Propagation of magnetization texture. (**a**), Static magnetic vortex pattern created by a radial current when $D$ = 0 mJ m^–2^. Scale bar, 100 nm. The same colour scale as in Fig. 1 shows the direction of magnetization. The inset shows a magnified image. (**b**), After changing from the state shown in (**a**) to $D$ = –1 mJ m^–2^, the magnetization state after 0.7 ns. Insets are intermediate images.

It can be seen that a non-zero DMI is essential for the wave to move (see Supplementary Note 6 for verification using simulations). This $V$ describes the velocity of a wave extending radially. To check this, it is assumed that a current is injected into the centre of the image and spreads in all directions (Fig. 3a). So, $\tau_{{\text{d}}}$ is proportional to $1/\left( {2{\uppi }\sqrt {x^{2} + y^{2} } } \right)$ because the current density is inversely proportional to the distance. Here, it was set so that $\left| {\tau_{{\text{d}}} } \right| = H_{k} /2$ at 42 nm from the centre (white dashed line in the inset of Fig. 3a). In addition, ${{\varvec{\upsigma}}}$ is also depicted in Fig. 3a. With $D = 0$, the initial uniform $+ \hat{z}$ magnetization state eventually converges to a stationary magnetization state (Fig. 3a). Because there is a strong $\tau_{{\text{d}}}$ (> $H_{k} /2$) near the centre, the vortex magnetization^42–44^ is formed along ${{\varvec{\upsigma}}}$. The magnetization is slightly fluctuating at the edge of the vortex, creating a circular wave shape. This state remains the same over time because DMI is zero (Fig. 3a). However, if we turn on the DMI at $t = 0$, the wave starts to move outward and then it generates concentric circle domains (Fig. 3b). As the waves spread, the effect of SOT becomes smaller, so the waves turn into magnetic domains having perpendicular ($\pm \hat{z}$) magnetization.

### Formation of the magnetic spiral

**Figure 4:**
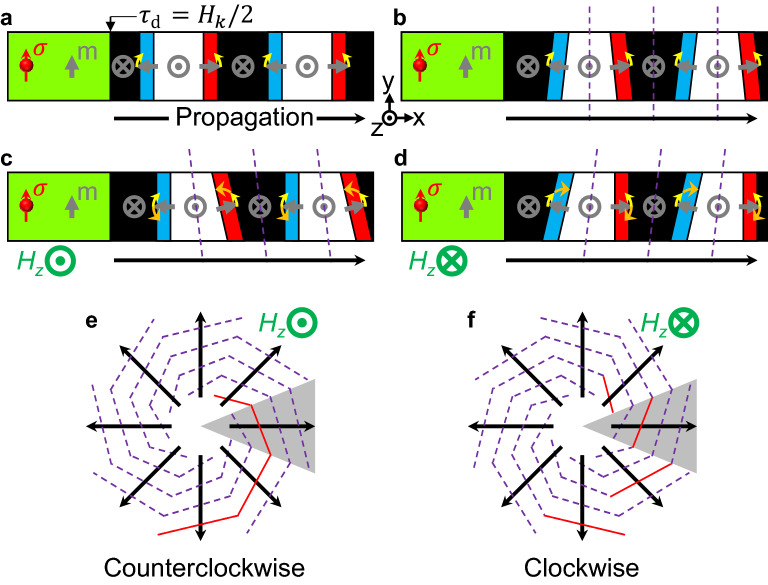
Tilting of magnetic domain wall and formation of a spiral. (**a**–**d**), The extended 1D model has a finite width. White and black represent magnetic domains with $\pm \hat{z}$ magnetization, and red and blue represent the domain wall areas with in-plane (*xy*-plane) magnetization. Because $\tau_{{\text{d}}}$ is strong in the green area, the magnetization is held in ${{\varvec{\upsigma}}}$. The grey arrows are the simplified magnetization vectors. Green arrows indicate applied external field directions. The yellow arrows indicate the torque generated by $\tau_{{\text{d}}}$, and the orange arrow indicates the torque generated by $H_{z}$. The purple dotted lines represent the overall slope of the magnetic texture. (**e**–**f**), spiral shapes created from the extended 1D model. The grey area means one extended 1D model.

We will now discuss the cause of the spiral shape. Situations where the extended 1D model has a finite width even on the *y*-axis are depicted in Fig. 4. From the left side to the right, $\tau_{{\text{d}}}$ gradually decreases, and at a specific position, $\tau_{{\text{d}}} = H_{k} /2$, a wave is generated (Fig. 4a). The generated wave changes to magnetic domains ($\pm \hat{z}$ magnetization) and magnetic domain walls ($\pm \hat{x}$ magnetization) and these domains and walls are pushed to the right ($+ \hat{x}$) by the SOT^28,45^. Note that, in Fig. 4, walls are simplified as having only $m_{x}$, but actual walls mainly have $m_{y}$. However, a chiral magnetization configuration consists of $m_{z}$ and small $m_{x}$ and results in SOT-induced unidirectional motions (see Supplementary Note 7 for the wall configuration in the 1D model). Previous studies show that the moving magnetic domain wall is tilted with respect to the direction of movement^15,46^. The SOT generates a torque of $- \gamma \tau_{{\text{d}}} {\mathbf{m}} \times \left( {{\mathbf{m}} \times {{\varvec{\upsigma}}}} \right)$ and this torque tries to rotate the wall magnetization (Eq. (1) and the yellow arrows in Fig. 4). The DMI causes the magnetic domain wall to have a stable structure (Fig. 4a)^41^. As the magnetization direction in the wall rotates, the slope of the magnetic domain wall also rotates (Fig. 4b). The important fact is that two adjacent walls tilt in opposite directions. Therefore, the tilting magnetic domain walls are likely to cancel each other, which is why concentric circle domains and walls are spread out (Fig. 3b). The dashed purple lines in Fig. 4b show the averaged slope of the domain walls, showing that the overall shape is not tilted. However, by adding a perpendicular magnetic field ($H_{z} \hat{z}$), we can choose the tilt direction (Fig. 4c,d). $H_{z}$ generates a torque by $- \gamma {\mathbf{m}} \times H_{z} \hat{z}$ and the torque direction always has the same direction of rotation in all domain walls (orange arrows in Fig. 4c,d). The effect of wall tilting due to $H_{z}$ remains between adjacent walls, resulting in tilting of the overall magnetization textures (dashed purple lines in Fig. 4c,d). When this tilting effect is applied to a rotationally symmetric structure, a spiral structure is formed (Fig. 4e,f). The grey-coloured area in Fig. 4e represents the extended 1D model described in Fig. 4a–d and the purple dotted lines represent the overall slope of the magnetic domain structure. Therefore, the perpendicular magnetic field determines the direction in which the spiral is wound.

### Hysteresis behaviour of the magnetic spiral

Once the winding direction of the spiral is determined, it has stability within a certain magnetic field. The red line in Fig. 4e represents a connected spiral arm. Tilting the purple dotted line as well as red lines shown in Fig. 4e in the opposite direction would cause the spiral to wind in the opposite direction. The result is shown in Fig. 4f, and it is important that the red lines no longer have a connected magnetization state. Therefore, if the magnetization state varies continuously, the winding direction of the spiral is assumed to have its own stability.

**Figure 5:**
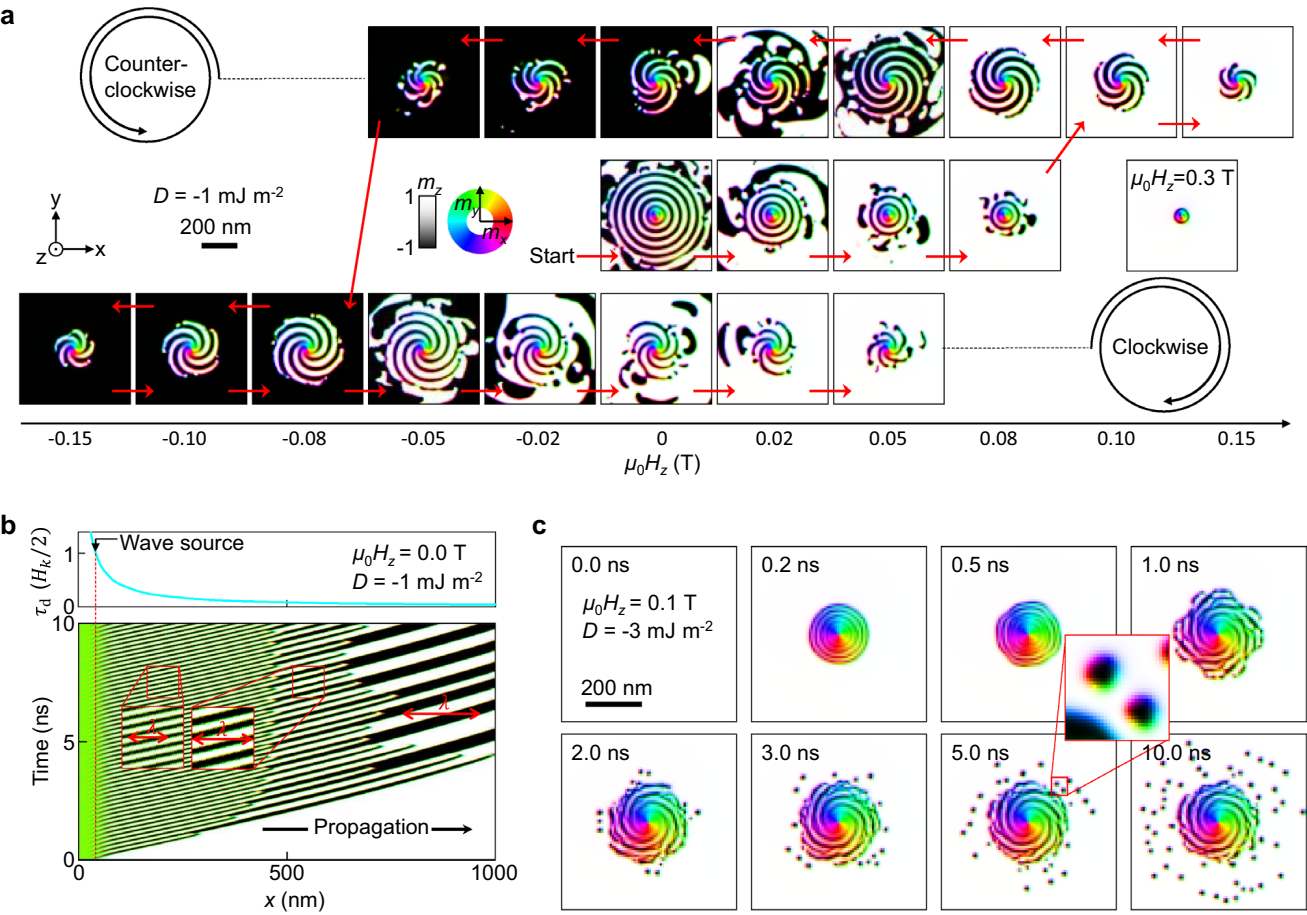
Hysteresis behaviour of magnetic spiral and application to skyrmion generation. (**a**), Change in stable magnetization states with the change in $H_{z}$. The red arrows indicate the order of $H_{z}$ change. (**b**), Change in magnetization states with time, as predicted from the 1D model. (**c**), An example of skyrmion creation. Scale bar, 200 nm.

The hysteresis in the spiral direction according to the change in the perpendicular magnetic field is shown in Fig. 5a. The images show a steady state that forms when the magnetic field changes gradually. In the initial ${\mathbf{m}} = \left( {0,0,1} \right)$ state, when $\tau_{{\text{d}}}$ is turned on with $\mu_{0} H_{z} = 0{\text{ T}}$, the concentric circle domains continue to spread. The domain state of concentric circles is maintained up to $\mu_{0} H_{z} = 0.08{\text{ T}}$, but the range of the concentric circles gradually decreases. When the field strength reaches 0.1 T, the concentric circle domain becomes a spiral domain. As expected in Fig. 4c and e, a counterclockwise (CCW) spiral domain is formed and rotates. This CCW spiral domain gradually decreases in area as the field increases. When a sufficient perpendicular magnetic field is applied ($\mu_{0} H_{z} = 0.3{\text{ T}}$), only the magnetic vortex structure remains in the centre. If the magnetic field is reduced in reverse, a CCW spiral is formed again, and the CCW spiral exists even when the sign of the magnetic field is reversed. If the magnetic field value is –0.08 T, the CCW spiral becomes a clockwise (CW) spiral. The CCW state does not go through the concentric circle state and immediately turns into the CW state because at $H_{z} < 0$ the CW spiral domain is preferred. This preference also increases the size of the spiral after CCW → CW switching. This CW spiral is maintained up to a magnetic field of 0.05 T even if the magnetic field direction is reversed again. In the simulations performed so far, the winding direction of the spiral tends to be preserved, so it can be expected that it will have hysteresis characteristics for the perpendicular magnetic field.

### Domain compression and destruction

Next, we discuss the phenomenon that the domain is compressed and disappears. All the spiral and concentric circle domains in Fig. 5a have a certain range in which their shape is maintained. This is due to a conservation law for a steady state. The result of solving the 1D model over time is shown in Fig. 5b. The initial ${\mathbf{m}} = \left( {0,0,1} \right)$ state and $\tau_{{\text{d}}} \propto 1/x$ with $\tau_{{\text{d}}} = H_{k} /2$ at $x = 42{\text{ nm}}$ are assumed. When $\tau_{{\text{d}}}$ is turned on at $t = 0{\text{ ns}}$, waves are continuously generated at $x = 42{\text{ nm}}$ (wave source). According to Eqs. (3) and (4), the generation frequency $f$ is expected to satisfy Eq. (5).

5 $$f = \frac{\omega }{2\pi } = - \frac{\gamma D}{{\pi \left( {1 + \alpha^{2} } \right)\mu_{0} M_{{\text{S}}} }}\sqrt {\frac{{K_{z} }}{2A}} .$$

This equation means that at the wave source, continuous $+ \hat{z}$ and $- \hat{z}$ domains are generated once every $1/f$ seconds. Therefore, if the whole system is to become a steady state, a set of $+ \hat{z}$ and $- \hat{z}$ domains must disappear somewhere at the same rate. In the simplest situation, they can be expected to disappear at the end of the magnetic material ($x = 1000{\text{ nm}}$ in Fig. 5b). However, the disappearance of domains in the middle region of the magnetic material occurs more often. In Fig. 5b, the set of domains generated from the source retain their shape up to 400 nm, and the set of domains disappear one by one at 400 nm. When the domain set disappears, the original wavelength ($\lambda = 2{\uppi }/k$) becomes longer and the domain density is reduced (insets of Fig. 5b). The same happens at the 700 nm position. It is well known that the speed of the domain wall increases with increasing $\tau_{{\text{d}}}$. So, the further from the wave source, the slower the magnetic domains and walls move. In this situation, the domains are continuously compressed in a certain space. Domains collapse and disappear and the rate at which domains and walls are pushed out does not keep up with the rate of domain creation. So, the spiral domains and the concentric circle domains have a finite size.

### Skyrmion creation

We will show skyrmion creation using the aforementioned phenomena. Because skyrmions can be treated as an ideal unit of information for applications, research on skyrmion generation is ongoing^24,47–50^. Most skyrmions have the shape of a circular domain in a PMA system^22–26^. In Fig. 5a, it can be seen that small domains exist at the boundary of the spiral and the concentric circle domains. This is because when a domain is compressed and disappears in two dimensions (2D), the domain undergoes the process of splitting into smaller fragments. The material parameters given in Fig. 5a cannot stabilize the skyrmion structure, so small fragment domains disappear over time. However, when the material values match the requirements for a stable skyrmion, skyrmions may form from the domain fragments. An example is shown in Fig. 5c. To stabilize the skyrmion, the intensity of the DMI was three times larger than the one used in Fig. 5a. All other parameters are the same as in Fig. 5a. When $H_{z}$ and $\tau_{{\text{d}}}$ are turned on in the initial ${\mathbf{m}} = \left( {0,0,1} \right)$ state, a spiral domain state is formed within 1 ns and fragments of domains are also formed at the edge of the spiral domain. These domain fragments transform into skyrmions over time (2–10 ns). An inset of Fig. 5c shows the skyrmions in detail that have downward magnetization cores and rotational symmetry. Importantly, domain fragments continue to be created at the edges of the spiral domain, so the number of skyrmions also increases over time. We believe that the spiral domain can be used to create skyrmions for spin devices.

## Discussions

We saw the wave magnetization forming where the size of the SOT is half the size of PMA. Previous studies have experimentally identified the appearance of skyrmions at the edges of the material and the formation of skyrmions in defects^47,51–53^. The defects and edges of the material may have property values ($M_{{\text{S}}}$, $K_{z}$, $A$) converging to zero, which reduces the PMA and increases the effect of SOT ($\propto 1/M_{{\text{S}}}$). Thus, our findings can provide another explanation for skyrmion creation on defects and edges^47,51–53^.

The magnetic spiral has a swirling in-plane magnetization near the centre, so it has a shape similar to the well-known magnetic vortex^43,54–57^. However, its dynamic characteristics are completely different to those of the vortex. For clarity, the discussion so far has assumed a situation in which all currents are injected into one point of the sample. If we consider the situation in which a uniform current is injected from a cylinder having a finite radius rather than a point contact, the influence of SOT by the radial current becomes zero exactly at the centre. In this case, a core with perpendicular magnetization at the centre of the spiral can be made. However, the dynamics of spiral domains (the rotating direction of spiral arms as well as winding directions) are not affected by the perpendicular cores (see Supplementary Note 8). This is different from the vortex dynamics, because the gyration of the vortex core strongly depends on the core polarity^43,54^. As regards chirality, it is important that the chirality of this vortex of the spiral is determined by sufficient SOT ($\left| {\tau_{{\text{d}}} } \right| > H_{k} /2$), selecting only a certain in-plane magnetization. Thus, the same SOT produces the same chirality of the vortex. We think it is not possible to reverse this chirality unless we change the current direction. This is different from previous results on the vortex that the chirality can be controlled by external means^55–57^.

In Fig. 5c, we showed that the skyrmion creation by the spiral domain. In addition to this, we can also think deletion of the skyrmion by a reversed current. This reversed current pushes skyrmions into the centre and destroys them (see Supplementary Note 9). We think this method of creation-deletion of skyrmion will be useful for skyrmion-based devices.

Through our study, it was found that the most important requirement for observing the spiral domain is the relative size of the SOT and the anisotropy energy, not the specific material parameters. The condition is written as $\left| {\tau_{d} } \right| > H_{k} /2$. So, a structure having lower PMA and larger SOT efficiency would be good. Current injection through point contacts would be best to increase the SOT strength, so it would be advantageous to inject the current through smallest possible area, such as SPM tips.

In this paper, we excluded the dipole-induced demagnetization energy to simplify explanation. In real magnetic systems, the demagnetization energy always exists and plays a major role in forming magnetization states. However, similar dynamics can be observed when considering the demagnetization energy (see Supplementary Note 10).

Our results related to skyrmion formation are similar to previous studies in that we use a spreading current to destroy the stripe domains to create the skyrmions^30^. However, it is different in that we have discovered an inevitable phenomenon of the LLG equation. That is, gradient of SOT can generate an oscillatory magnetization state when it passes $\left| {\tau_{d} } \right| = H_{k} /2$.

## Conclusion

We found the conditions under which a magnetic spiral domain is formed from the basic magnetic dynamics equation. This magnetic spiral can control the winding direction relative to the external magnetic field, unlike any other spiral in nature. These magnetic spirals can create skyrmions, which could be used to develop spin devices. Our through understanding on this magnetic spiral structure will inspire the understanding other spirals in nature^1^.

## Method

Mumax3 was used to perform the simulation^38^. This program predicts magnetic dynamics by applying the LLG equation to a magnetic system composed of discrete cells. The following values were used for the material parameters (unless otherwise specified): $\gamma$ = 2.21 × 10^5^ rad m A^–1^ s^–1^; $\mu_{0}$ = 4π × 10^–7^ T m A^–1^; $M_{{\text{S}}}$ = 1 × 10^6^ A m^–1^; $A$  = 1 × 10^–11^ J m^–1^; $K_{z}$ = 5 × 10^5^ J m^–3^; $\mu_{0} H_{k}$ = 1 T; $D$ = –1 × 10^–3^ J m^–2^; $\alpha$ = 0.1. The dipole–dipole interaction was ignored. For Fig. 1, $\mu_{0} H_{z}$ = 0.1 T. In all simulations, the size of the unit cell was fixed at 2 × 2 × 1 nm^3^. The assumed grid size varied according to whether it is a 1D or a 2D model, and also on models used in each figures. Because the assumption involved a thin film situation, there is only one cell in the *z*-direction. For Figs. 1, 3a,b, $\left( {N_{x} ,{ }N_{y} , N_{z} } \right)$ = (400, 400, 1), where $N_{i}$ is the number of cells on the *i*-axis. For Fig. 2a,b, $\left( {N_{x} ,{ }N_{y} , N_{z} } \right){ }$ = (500, 1, 1). For Fig. 5a, $\left( {N_{x} ,{ }N_{y} , N_{z} } \right)$ = (600, 600, 1). For Fig. 5b, $\left( {N_{x} ,{ }N_{y} , N_{z} } \right)$ = (500, 1, 1). For Fig. 5c, $\left( {N_{x} ,{ }N_{y} , N_{z} } \right)$ = (600, 600, 1). The position-dependent ${{\varvec{\upsigma}}} = \left( {\frac{ - y}{{\sqrt {x^{2} + y^{2} } }},\frac{x}{{ \sqrt {x^{2} + y^{2} } }}, 0} \right)$ was used in Figs. 1, 3a, 3b, 5a, and 5c. A uniform ${{\varvec{\upsigma}}} = \left( {0,1,0} \right)$ was used for Figs. 2a, 2b, and 5b. It was set so that $\left| {\tau_{{\text{d}}} } \right| = H_{k} /2$ at 42 nm from the centre for all 2D models. The temperature was assumed to be 0 K. The stationary states shown in Figs. 2a, 2b, and 3a were assumed from the initial states to the states after 50 ns.

## Supplementary Information

Supplementary Information 1.

Supplementary Video 1.
