# Supplementary material for: Reversible magnetic spiral domain
Source: Sci Rep. 2021 Oct 25;11:20970. doi: 10.1038/s41598-021-00016-z (PMC8546083; doi:10.1038/s41598-021-00016-z)
Supplement: Supplementary file 1 — Supplementary Information 1. [file 41598_2021_16_MOESM1_ESM.docx]

**Supplementary Information for**

**Reversible magnetic spiral domain**

Kyoung-Woong Moon, Seungmo Yang, and Chanyong Hwang*

*Quantum Spin Team, Korea Research Institute of Standards and Science, Daejeon 34113, Republic of Korea*

*Email: cyhwang@kriss.re.kr

This file includes:

Supplementary Note 1-10

Supplementary Figure 1-11

**Supplementary Note 1. Stationary state of uniform magnetization**

We will solve $\mathbf{m}\times\mathbf{H}_{\mathrm{eff}}=-\tau_{d}\mathbf{m}\times\left( \mathbf{m}\times\boldsymbol{\sigma} \right)$ step-by-step with $\mathbf{m=(}m_{x}, m_{y},m_{z})$, $\mathbf{H}_{\mathrm{eff}}=H_{k}m_{z}\hat{z}$, and $\boldsymbol{\sigma}=+\hat{y}$. Cross products of vectors are solved as follows.

$$\left| \begin{matrix} \hat{x} & \hat{y} & \hat{z} \\ m_{x} & m_{y} & m_{z} \\ 0 & 0 & H_{k}m_{z} \end{matrix} \right|=-\tau_{d}\left| \begin{matrix} \hat{x} & \hat{y} & \hat{z} \\ m_{x} & m_{y} & m_{z} \\ -m_{z} & 0 & m_{x} \end{matrix} \right|. (S1)$$

Then, we get 3 equations.

$$H_{k}{m_{y}m}_{z}=\boldsymbol{-}\tau_{d}m_{x}m_{y}, (S2a)$$

$$-H_{k}{m_{x}m}_{z}=\tau_{d}\left( m_{x}^{2}+m_{z}^{2} \right), (S2b)$$

$$0=\boldsymbol{-}\tau_{d}m_{y}m_{z}. (S2c)$$

Eq. (S2c) means $\tau_{d}=0$ or $m_{y}m_{z}=0$. $\tau_{d}=0$ case is trivial because $\boldsymbol{m=\pm}\hat{z}\boldsymbol{=}(0,0,\pm1)$ due to the PMA ($H_{k}>0$). Now, we will think about only $m_{y}m_{z}=0$. We will divide it again into $m_{y}=0$ and $m_{z}=0$.

When $m_{y}=0$, Eq. (S2b) becomes $-H_{k}{m_{x}m}_{z}=\tau_{d}$ because we already know $m_{x}^{2}+m_{y}^{2}+m_{z}^{2}=1$ (property of unit vector). In addition, from $m_{x}^{2}+m_{z}^{2}=1$, it can be written as $m_{z}=\cos\theta$ and $m_{x}=\sin\theta$. Here, $\theta$ means the angle between +$\hat{z}$ axis and $\mathbf{m}$. If the initial magnetization $\mathbf{m}=\mathbf{+}\hat{z}$ at $\tau_{d}=0$, increasing $\tau_{d}$ tilts $\mathbf{m}$ to $\mathbf{-}\hat{x}$. This is because $\tau_{d}>0$ and $-H_{k}{m_{x}m}_{z}=\tau_{d}$ forces $m_{x}$ and $m_{z}$ to have opposite signs. This magnetization tilting to $\mathbf{-}\hat{x}$ continues within the range of $0\leq\tau_{d}\leq H_{k}/2$. When $\tau_{d}=H_{k}/2$, the magnetization angle is inclined by -45°. In other words, $\mathbf{m}=(-\frac{1}{\sqrt{2}},0,\frac{1}{\sqrt{2}})$ and $\sin\theta\cos\theta=-1/2$. If $\tau_{d}$ exceeds $H_{k}/2$, there is no possible $\theta$ because $-1/2\leq\sin\theta\cos\theta\leq+1/2$ (property of trigonometric functions). Thus, we have to think about the $m_{z}=0$ case.

When $m_{z}=0$, Eq. (S2a) implies that $\tau_{d}m_{x}m_{y}=0$. We're already dealing with a situation where $\tau_{d}\neq0$, so either $m_{x}=0$ or $m_{y}=0$. If $m_{y}=0$, this means $\mathbf{m}=(\pm1,0,0)$. However, at the same time, Eq. (S2b) becomes $0=\tau_{d}m_{x}^{2}$. So, $\mathbf{m}=(\pm1,0,0)$ situation cannot exist. The last situation left is $m_{x}=0$ with $m_{z}=0$. In other words, $\mathbf{m}=(0,\pm1,0)$ and this magnetization satisfies all Eq. (S2). But, considering the direction of the SOT torque, it can be seen that when $\tau_{d}>0$, the torque that aligns $\mathbf{m}$ in the direction of $\boldsymbol{\sigma}$ always occurs (Supplementary Figure 1). So, at $\tau_{d}>H_{k}/2$, $\mathbf{m}$ will be aligned in $\boldsymbol{\sigma}$. Note that, $\mathbf{m}=(0,1,0)$ always satisfies Eq. (S2) even if $\tau_{d}<H_{k}/2$. But, considering that there is no current as the starting state, the $\mathbf{m}=(0,1,0)$ state is difficult to access when $\tau_{d}<H_{k}/2$.

In short, if we think about only $\tau_{d}\geq0$, $\mathbf{m}=\pm\hat{z}$ state is preferred at $\tau_{d}=0$. These perpendicular magnetizations persist up to $\tau_{d}=H_{k}/2$ with slightly rotated magnetization from $\boldsymbol{\pm}\hat{z}$ to $\boldsymbol{\mp}\hat{x}$. This magnetization rotation keeps $m_{x}m_{z}=-\tau_{d}/H_{k}$ and $m_{y}=0$. So, the maximum tilted state is $\boldsymbol{m=(\pm}\frac{1}{\sqrt{2}},0,\mp\frac{1}{\sqrt{2}})$. When $\tau_{d}>H_{k}/2$, only $\mathbf{m}\boldsymbol{=}\mathbf{+}\hat{y}$ is possible. Therefore, a sudden magnetization jump from $\boldsymbol{m=(\pm}\frac{1}{\sqrt{2}},0,\mp\frac{1}{\sqrt{2}})$ to $\mathbf{m=}(0,1,0)$ occurs at $\tau_{d}=H_{k}/2$.


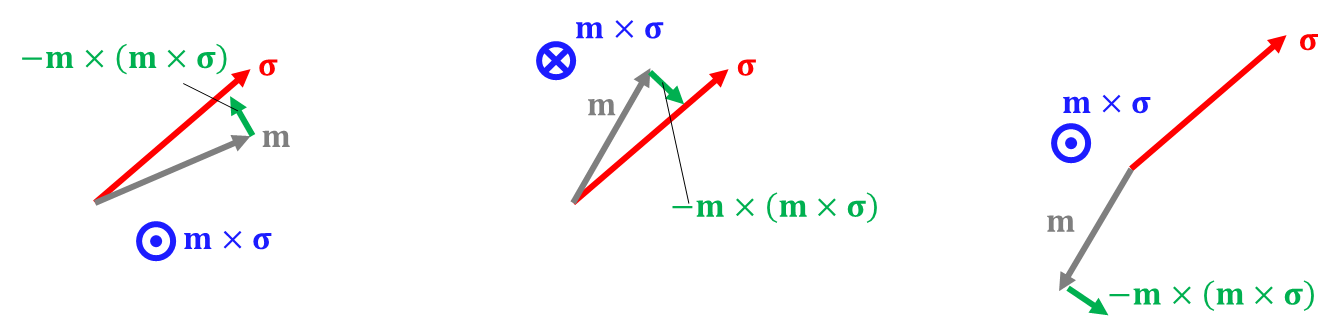


Supplementary Figure 1 | Directions of SOT torque for $\boldsymbol{\tau}_{\mathbf{d}}\boldsymbol{>0}$. $\mathbf{m}$ tries to be aligned in the direction of $\boldsymbol{\sigma}$.

**Supplementary Note 2.** **Stabilized magnetization states in 1D model**

As reinforcement data for Fig. 2a, b, magnetization states generated when initiated from a uniform magnetization state ($\mathbf{m}=(0,0,1)$) rather than from a random initial state was obtained (Supplementary Figure 2). The results are that a wave magnetization is still produced at the $\tau_{d}=H_{k}/2$ location. We also confirmed that the same $k$ is formed at $\tau_{d}=H_{k}/2$ positions even when performing simulations that doubled the size of the cell size. This shows that $k$ is a value determined by given material parameters.

**
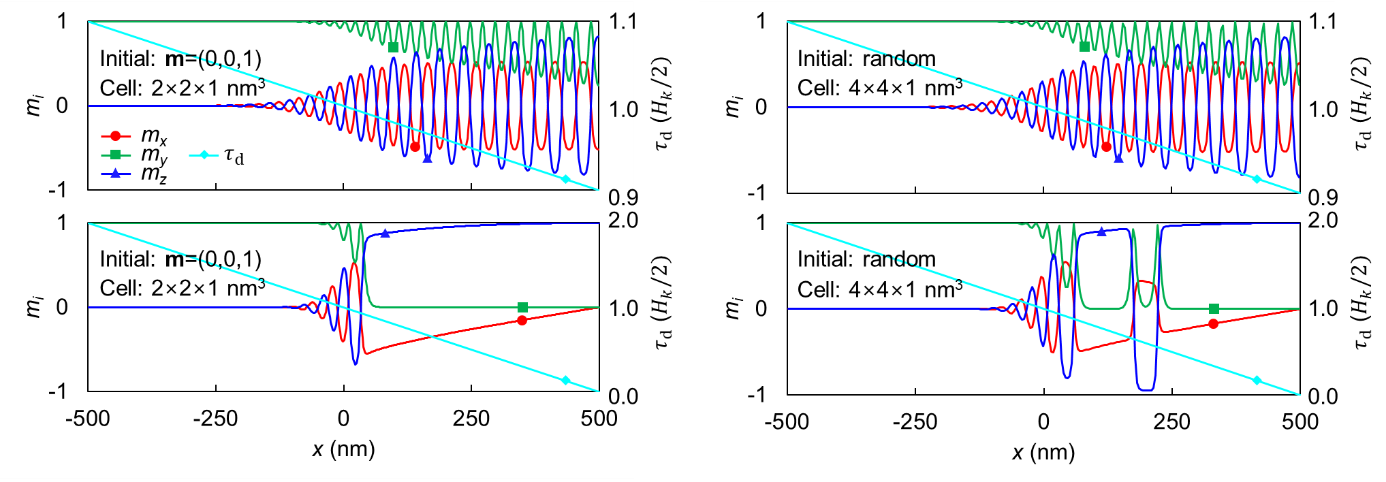
Supplementary Note 3. Stable magnetization states**

Supplementary Figure 2 | Results of an initial uniform state and results of a larger cell size. The simulation conditions are the same as those shown in Figs. 2a and 2b, except for the specified values shown on the graph.

We use the 1D model with $\left( N_{x}, N_{y}, N_{z} \right)$= (8000, 1, 1) and a cell size of 4 × 4 × 1 nm^3^. The range of $x$ coordinates is from $-16 \mu m$ to $+16 \mu m$. Linearly varying $\tau_{d}$ on the *x*-axis was assumed with $\tau_{d}=H_{k}$ at $x=-16 \mu m$ and $\tau_{d}=0$ at $x=+16 \mu m$. All other parameters are the same as those shown in Fig. 2a and 2b. Three different initial states were assumed at time 0. Two initial states are $\mathbf{m=}(0,0,1)$ and random state. The third initial state is described by $-H_{k}{m_{x}m}_{z}=\tau_{d}$ with $m_{y}=0$ where $0\leq\tau_{d}\leq H_{k}/2$ and $m_{y}=1$ where $\tau_{d}>H_{k}/2$, which are solutions described in Supplementary Note 1. When the initial state is $\mathbf{m=}(0,0,1)$, the magnetization states are stabilized as shown in Supplementary Fig. 3.


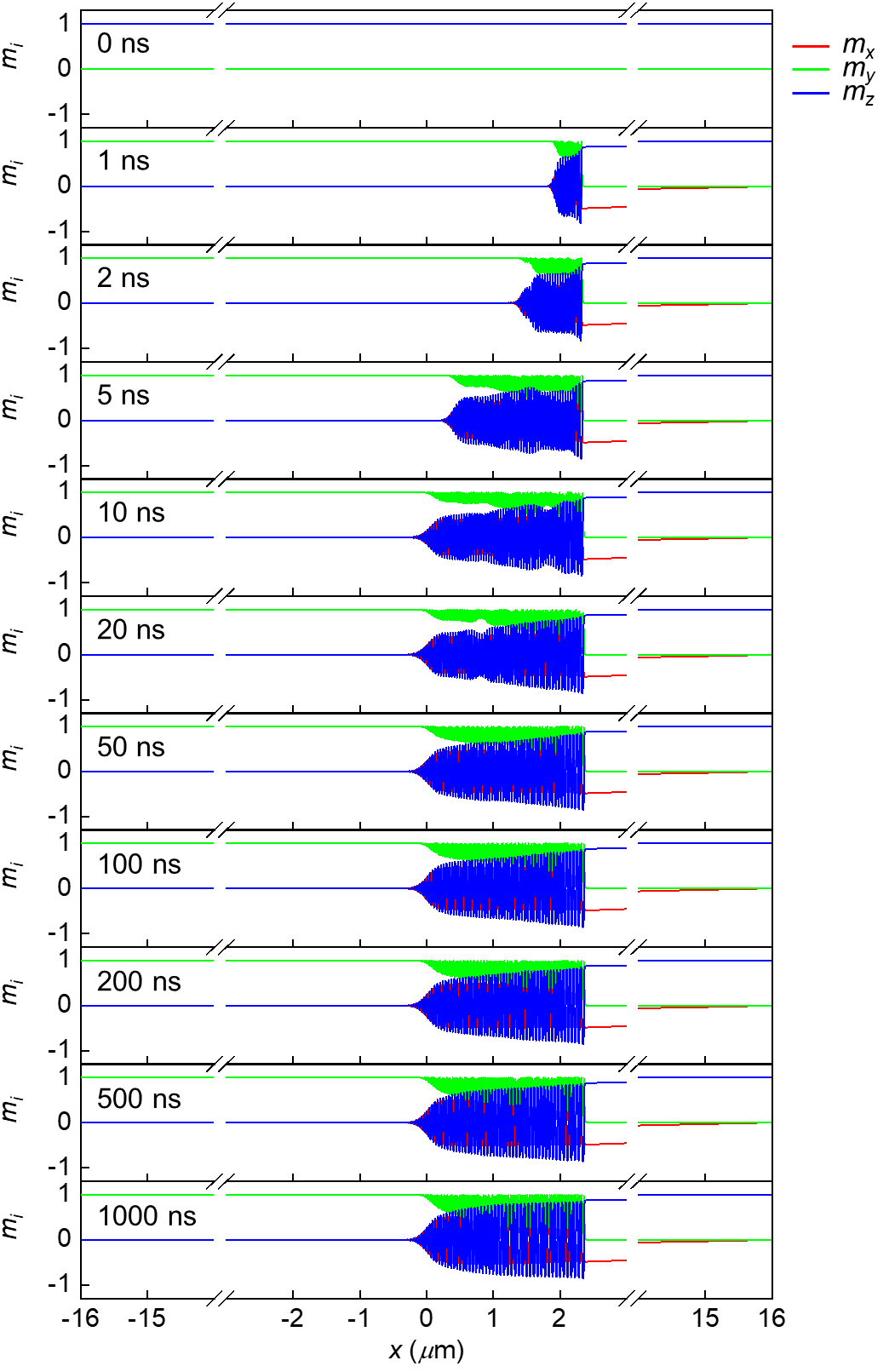


**Supplementary Figure 3 | Stabilization of the magnetization state.**

The initial state is immediately divided into one state of $m_{z}\sim1$ and the other state of $m_{y}\sim1$ on the basis of $x\sim2.5 \mu m$, and an oscillatory magnetization is formed between them. Then, such oscillation extends to $x\sim0 \mu m$ within 10 ns. Note that the almost uniform $m_{z}$~1 state is always stable in the region $x>2.5 \mu m$.

For the random initial state, the results are shown in Supplementary Fig. 4. The stabilization of oscillations of magnetization near $x\sim0 \mu m$ is the same as the previous result. But, even in the $x>2.5 \mu m$ region, it can be seen that the states of $m_{z}\sim1$ and $m_{z}\sim-1$ are always mixed and almost stably present. Where the position is near $x=15 \mu m$, we can see that domains slowly disappear one by one.


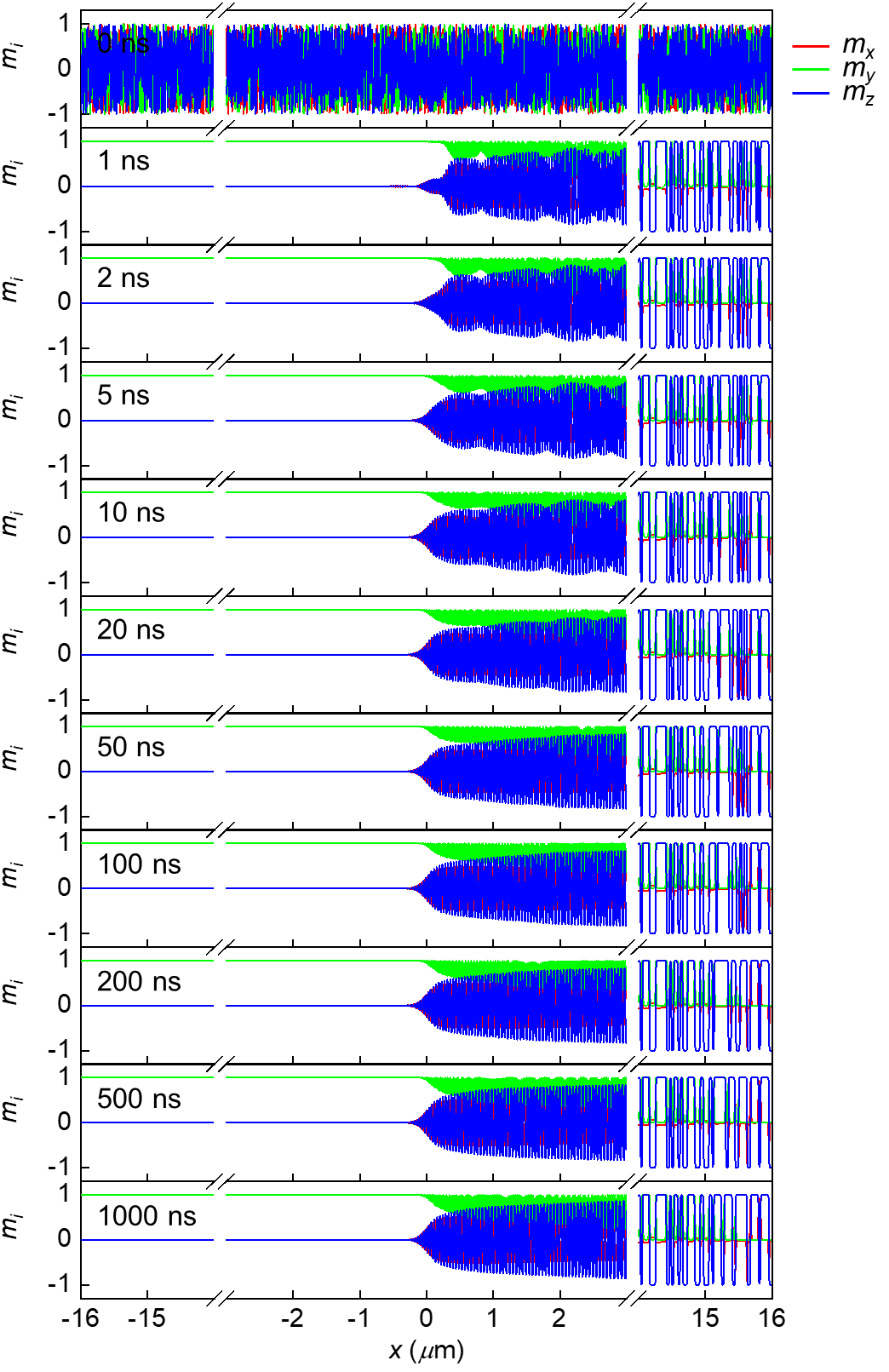


**Supplementary Figure 4 | Stabilization of the magnetization state.**

Supplementary Figure 5 shows the result of the third initial condition. The magnetization oscillation is immediately formed at $x=0 \mu m$. The region where the oscillation exists extends very slowly to the region where $x>0 \mu m$.


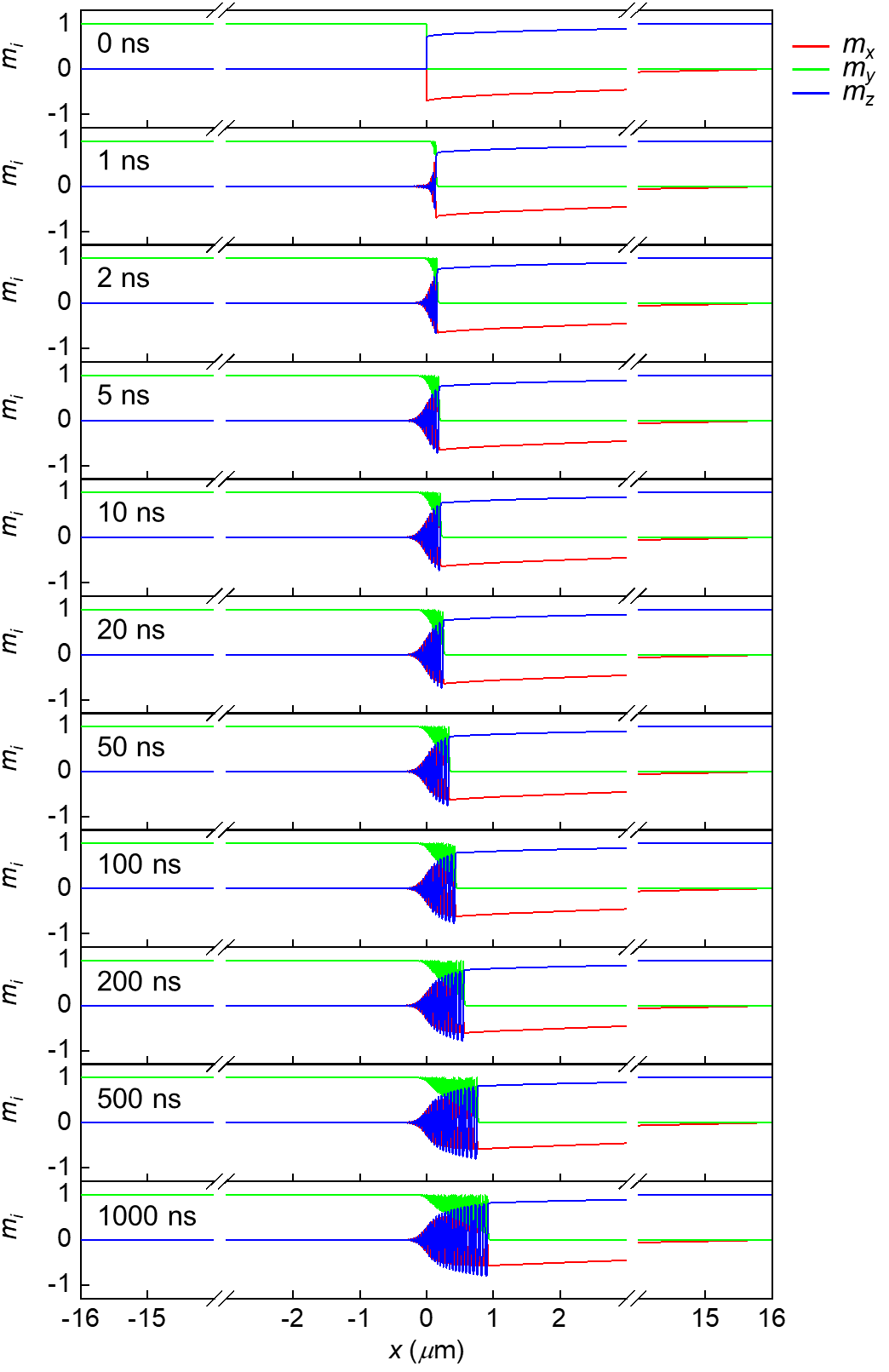


**Supplementary Figure 5 | Stabilization of the magnetization state.**

Through the above simulations, we expect the phase diagram to be as follows. First, $\tau_{d}>H_{k}/2$ makes $\mathbf{m=}(0,1,0)$. Second, $\tau_{d}=H_{k}/2$ tends towards an oscillatory magnetization and such oscillation expands its range into the region $\tau_{d}<H_{k}/2$, but it slows down rapidly. Third, $\tau_{d}<H_{k}/2$ tends towards the uniform $m_{z}\sim1$ or $m_{z}\sim-1$. But even if the two states are mixed, they can exist stably.

In this way, we can get a rough understanding of the phase diagram. However, it is important to note that all magnetization vectors are changing continuously in space, so the oscillatory states and uniform magnetization states are also changing continuously. Therefore, clearly dividing the boundaries of each region should be difficult. For example, Supplementary Fig. 6 shows a detailed magnetization state after 1000 ns when the run started with initial random state. Oscillatory magnetization states having a constant period can be seen in the $4\sim6 \mu m$ region, but it becomes more and more difficult to distinguish whether it is an oscillation form or a wide domain at $x>6 \mu m$.


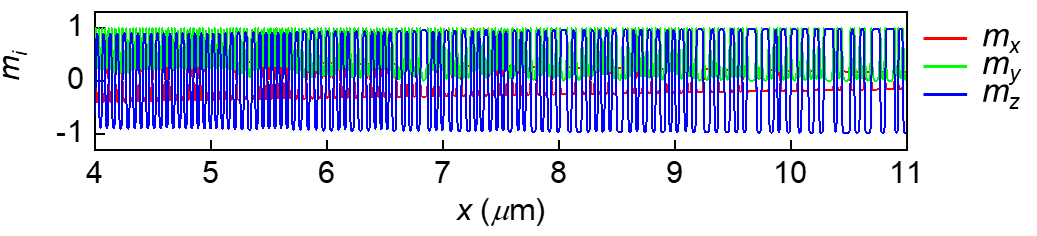


**Supplementary Figure 6 | The magnetization state at 1000 ns.**

**Supplementary Note 4.** **The process of deriving** $\boldsymbol{k}$

We will solve $\mathbf{m}\times\mathbf{H}_{\mathrm{eff}}=-\tau_{d}\mathbf{m}\times\left( \mathbf{m}\times\boldsymbol{\sigma} \right)$ step-by-step with $\mathbf{m=(}m_{x}, m_{y},m_{z})$, $\mathbf{H}_{\mathrm{eff}}=A^{*}{\partial^{2}\mathbf{m}}/{\partial x^{2}}+H_{k}m_{z}\hat{z}$, and $\boldsymbol{\sigma}=+\hat{y}$. Cross products of vectors are solved as follows. Here, $m_{i}^{''}={\partial^{2}m_{i}}/{\partial x^{2}}$.

$$\left| \begin{matrix} \hat{x} & \hat{y} & \hat{z} \\ m_{x} & m_{y} & m_{z} \\ A^{*}m_{x}'' & A^{*}m_{y}^{''} & A^{*}m_{z}^{''}+H_{k}m_{z} \end{matrix} \right|=-\tau_{d}\left| \begin{matrix} \hat{x} & \hat{y} & \hat{z} \\ m_{x} & m_{y} & m_{z} \\ -m_{z} & 0 & m_{x} \end{matrix} \right|. (S3)$$

Then, we get 3 equations.

$$A^{*}m_{y}m_{z}^{''}-A^{*}m_{z}m_{y}^{''}+H_{k}{m_{y}m}_{z}=\boldsymbol{-}\tau_{d}m_{x}m_{y}, (S4a)$$

$$A^{*}m_{z}m_{x}^{''}-A^{*}m_{x}m_{z}^{''}-H_{k}{m_{x}m}_{z}=\tau_{d}(m_{x}^{2}+m_{z}^{2}), (S4b)$$

$$A^{*}m_{x}m_{y}^{''}-A^{*}m_{y}m_{x}^{''}=\boldsymbol{-}\tau_{d}m_{y}m_{z}. (S4c)$$

Inserting $\mathbf{m=}\left( X\sin kx,1,-X\sin kx \right)$ and using $m_{y}^{''}=0$ and $m_{y}=1$ change above equations as follows.

$$k^{2}A^{*}X-H_{k}X=\boldsymbol{-}\tau_{d}X, (S5a)$$

$$k^{2}A^{*}X^{2}-k^{2}A^{*}X^{2}+H_{k}X^{2}=\tau_{d}(X^{2}+X^{2}), (S5b)$$

$$k^{2}A^{*}X=\tau_{d}X. (S5c)$$

From Eq. (S5b), $H_{k}=2\tau_{d}$. Substituting this into Eq. (S5c), we get $k^{2}={\tau_{d}}/{A^{*}}={H_{k}}/{(2A^{*})}$.

**Supplementary Note 5.** **The process of deriving** $\boldsymbol{V}$

We will solve $\dot{\mathbf{m}}=-\gamma\mathbf{m}\times\mathbf{H}_{\mathrm{eff}}+\alpha\mathbf{m}\times\dot{\mathbf{m}}\boldsymbol{-}\gamma\tau_{d}\mathbf{m}\times\left( \mathbf{m}\times\boldsymbol{\sigma} \right)$ step-by-step with $\mathbf{m=(}m_{x}, m_{y},m_{z})$, $\mathbf{H}_{\mathrm{eff}}=A^{*}{\partial^{2}\mathbf{m}}/{\partial x^{2}}+D^{*}\hat{y}\times({\partial\mathbf{m}}/{\partial x})+H_{k}m_{z}\hat{z}$, and $\boldsymbol{\sigma}=+\hat{y}$. Firstly, the time derivative terms are collected on the left side of the equation.

$\left( 1+\alpha^{2} \right)\dot{\mathbf{m}}=-\gamma\mathbf{m}\times\mathbf{H}_{\mathrm{eff}}-\alpha\gamma\boldsymbol{m\times}\left( \boldsymbol{m\times}\mathbf{H}_{\mathrm{eff}} \right)\boldsymbol{-}\gamma\tau_{d}\mathbf{m}\times\left( \mathbf{m}\times\boldsymbol{\sigma} \right)+\alpha\gamma\tau_{d}\mathbf{m}\times\boldsymbol{\sigma}\boldsymbol{.}(S6)$

If you put $\mathbf{m}=\left( X\sin(kx-\omega t),1,-X\sin(kx-\omega t) \right)$ on the left side of Eq. (S6), it is as follows.

$$-\omega\left( 1+\alpha^{2} \right)\left( XC,0,-XC \right), (S7)$$

where, $C=\cos(kx-\omega t)$. In the same way, $S=\sin(kx-\omega t)$. The right side of Eq. (S6) is,

$$\gamma\left( \begin{matrix} -X\left[ \begin{aligned} -S\left\{ \alpha X^{2}S^{2}\left( A^{*}k^{2}-H_{k} \right)-\tau_{d} \right\} \\ +S\left( A^{*}k^{2}-H_{k}-\alpha\tau_{d} \right)+\alpha D^{*}kC\left( X^{2}S^{2}+1 \right) \end{aligned} \right], \\ X^{2}\left[ \begin{aligned} S^{2}\left( A^{*}k^{2}-H_{k} \right)+S^{2}\left( \alpha A^{*}k^{2}-\alpha H_{k}+\tau_{d} \right) \\ +\alpha D^{*}kSC-D^{*}kSC+\tau_{d}S^{2} \end{aligned} \right], \\ X\left[ \begin{aligned} S\left\{ \alpha X^{2}S^{2}\left( A^{*}k^{2}-H_{k} \right)+\alpha A^{*}k^{2}-\alpha H_{k}+\tau_{d} \right\} \\ +\alpha S\left( -D^{*}kX^{2}SC+\tau_{d} \right)-D^{*}kC \end{aligned} \right] \end{matrix} \right). (S8)$$

Since we want to know the average rate at which the waveform moves while maintaining its overall shape, we perform the dot product of ${\partial\mathbf{m}}/{\partial x}$ on Eq. (S7) and Eq. (S8), and we integrate over one period. Finally, we get,

$$-\pi\omega X^{2}\left( 1+\alpha^{2} \right)=\pi\gamma D^{*}kX^{2}. (S9)$$

**Supplementary Note 6. Verification of characteristics of** $\boldsymbol{k}$ **and** $\boldsymbol{\omega}$ **with simulations**

We perform 1D simulations to verify that the wave characteristics presented in Equations (3) and (4). $(N_{x}, N_{y}, N_{z})$=(500,1,1) is used, where $N_{i}$ is the number of cells on the $i$-axis. Material parameters are described in Method section. Uniform SOT is assumed such as $\boldsymbol{\sigma}=(0,1,0)$ and $\tau_{d}=H_{k}/2$ to see the pure characteristics of the wave source. The periodic boundary condition is introduced in the *x* direction to avoid situations where waves meet the material boundary. Initially, assuming a random magnetization state, the waveform is formed and propagates after a certain time. By saving changes in the magnetization state for a period of time and performing FFT, information about $k$ (wave number) and $\omega$ (angular frequency) can be obtained. Supplementary Figure 7 shows $k$ and $\omega$ as a function of $D$. In Equations (3) and (4), $k$ is not dependent on $D$ and $\omega$ is expected to depend linearly on $D$, and simulations show the same results. The larger the DMI, the more severe the overlapping of various waves (inset of Supplementary Figure 7), thereby widening the spectrum of $k$ and $\omega$, but the general trend fit well with the expected values in the equations.


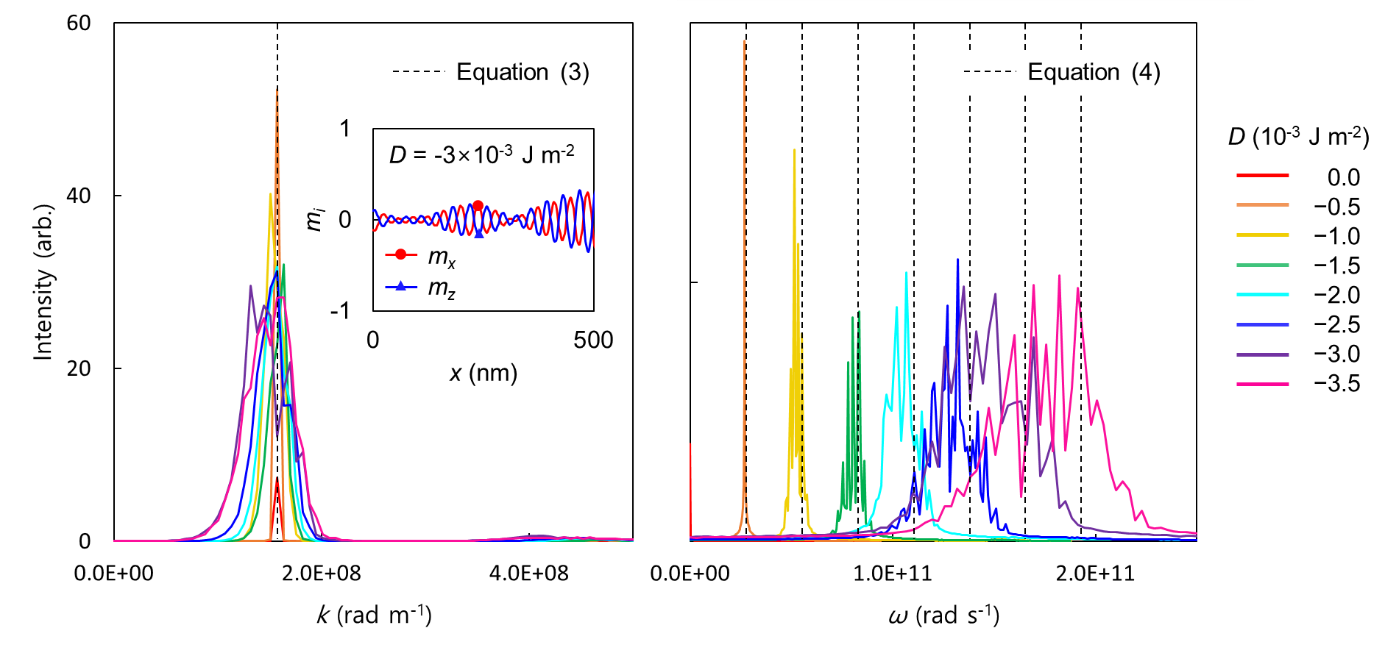


**Supplementary Figure 7 |** $\boldsymbol{D}$ **dependence of** $\boldsymbol{k}$ **and** $\boldsymbol{\omega}$ **obtained by simulations.** The solid lines are the values obtained from the simulations and the dotted lines are the values expected from equations. The inset shows wave patterns with large $D$.

**Supplementary Note 7. Magnetization configuration in the 1D model.**

We use $\left( N_{x}, N_{y}, N_{z} \right)$= (500, 1, 1). The unit cell is 2 × 2 × 1 nm^3^; $\boldsymbol{\sigma}=(0,1,0)$; and $\tau_{d}\propto1/x$ with $\tau_{d}=H_{k}/2$ at $x=42 nm$ are assumed. $D$=$-$1 mJ/m^2^. All other parameters are described in Method. Initial magnetization is $\mathbf{m}=(0,0,1)$. The magnetization state after 10 ns is shown in Supplementary Fig. 8. We can see magnetic domains and domain walls in $x>42 nm$. The domain walls have small $m_{x}$ but the signs of $m_{x}$ of adjacent walls are opposite to each other. So, $m_{x}$ of walls and $m_{z}$ of domains form chiral magnetization states such as (+*z*)-(+*x*)-(-*z*)-(-*x*)-(+*z*). In the main text, we exaggerated these chiral states to allow us to describe them in simple terms. It is notable that the main mechanism for the spiral formation is the rotation of the domain wall due to the rotation of the wall magnetization. This mechanism is still valid for the chiral states with small amplitudes because the rotational direction of the wall magnetization by $\tau_{d}$ and $H_{z}$ is maintained.


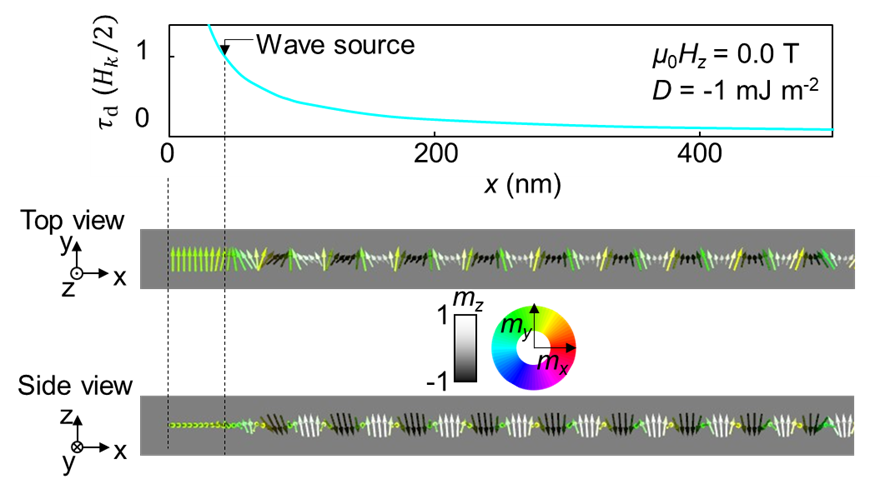


**Supplementary Figure 8 | Detailed magnetization configuration in the 1D model.**

**Supplementary Note 8. Finite size of current injection and core of spiral domain.**

Considering the situation that the uniform current is injected from a cylindrical electrode with a finite radius instead of at a point contact, the current density should be zero at the centre (Supplementary Figure 9a). This makes zero SOT at the centre, so there should be a perpendicular magnetization as a spiral core.

We applied this modified current density with 20 nm of the cylinder radius to the same situation of Fig. 1. In addition to this, different initial magnetization states (uniform $+\hat{z}$ and uniform $-\hat{z}$) induce opposite magnetization of the perpendicular cores (Supplementary Fig. 9b). However, the dynamics of the spiral domain (the rotating direction of spiral arms as well as winding directions) are not affected by the perpendicular cores.


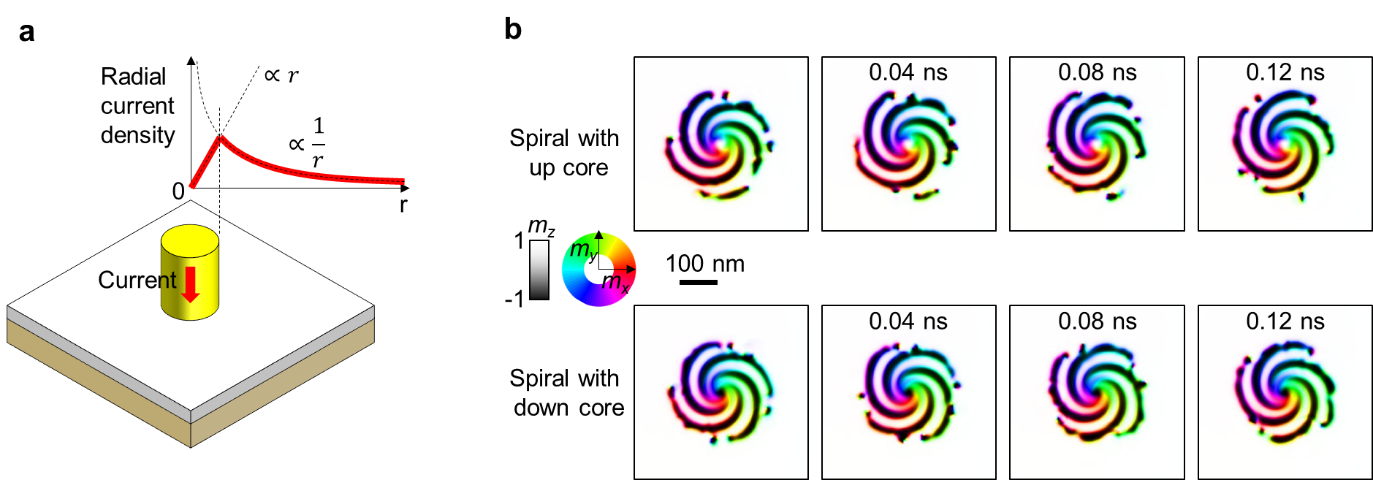


**Supplementary Figure 9 | Current injection from the cylindrical electrode.** **a**, Distribution of radial current density in the heavy metal layer. **b**, Dynamics of spiral domain with opposite cores. Simulation conditions are the same as those of Fig. 1 except for the current density distribution.

**Supplementary Note 9. Deletion of skyrmions.**

Following Supplementary Fig. 10 shows deletion of skyrmions. After creation of skyrmions shown in Fig. 5c, we turned off the current and waited for 10 ns. Then, we turned on the reversed current. The current pulls the magnetization states (including skyrmions) into the centre and destroys them. So, the number of skyrmions decreases over time.


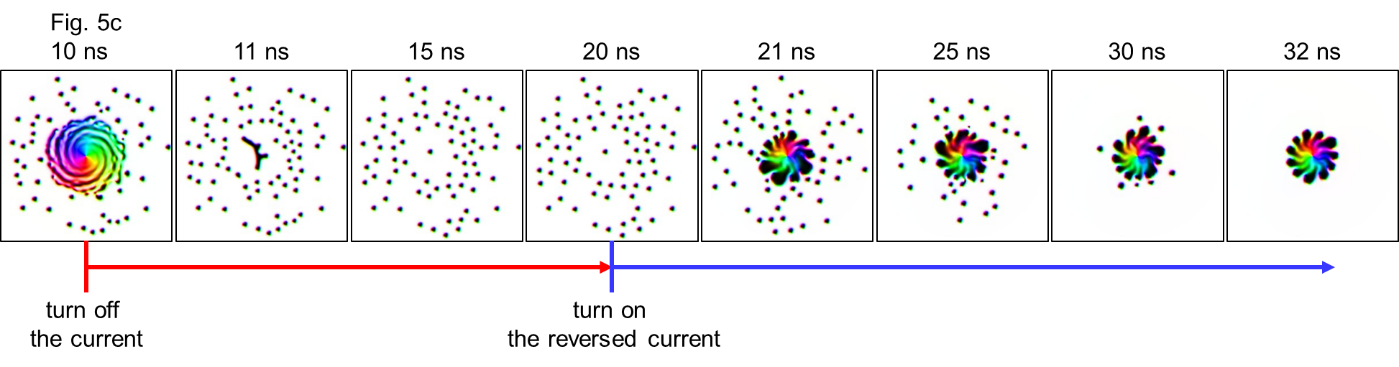


**Supplementary Figure 10 | Deletion of skyrmions.** The magnetization state shown in Fig. 5c is relaxed without current for 10 ns. Then, reversed current is turned on.

**Supplementary Note 10. Effect of demagnetization energy.**

The demagnetization energy in the thin film structure is often approximated to the extent of adjusting the value of the perpendicular anisotropy. The simplest form is $K_{\mathrm{eff}}=K_{z}-\frac{\mu_{0}}{2}M_{S}^{2}$ and the effective energy to hold the perpendicular magnetization is determined by $K_{\mathrm{eff}}$. To confirm this, we use $K_{z}$=1.12831×10^6^ J/m^3^ (so $K_{\mathrm{eff}}$=0.5×10^6^ J/m^3^) with the demagnetization energy. Similar results to those shown in Fig. 1 were produced using this condition including the formation of a similar spiral formed (Supplementary Fig. 11). The actual demagnetization effect is nonlocal and the up and down magnetizations tend to mix more due to this demagnetization. Thus, a larger spiral is formed.


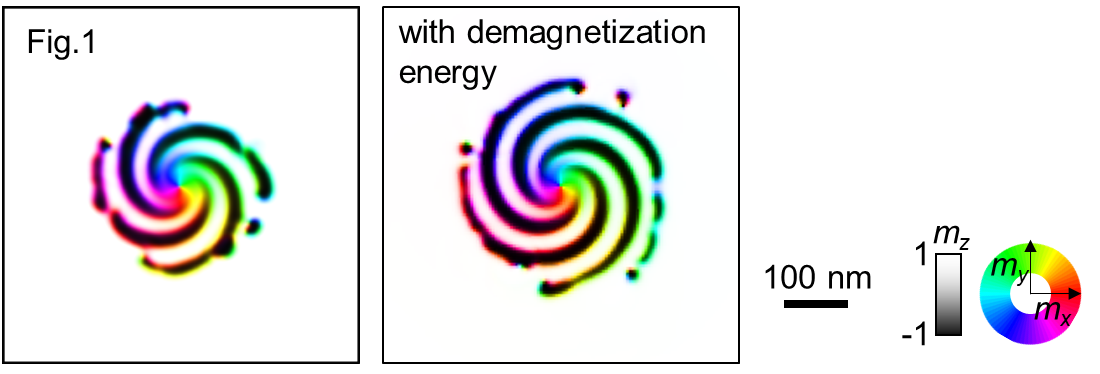


**Supplementary Figure 11 | Spiral with demagnetization energy.**
